# Maximum Stress Estimation Model for Multi-Span Waler Beams with Deflections at the Supports Using Average Strains

**DOI:** 10.3390/s150407728

**Published:** 2015-03-30

**Authors:** Sung Woo Park, Byung Kwan Oh, Hyo Seon Park

**Affiliations:** 1 Center for Structural Health Care Technology in Buildings, Yonsei University, Seoul 120-749, Korea; E-Mail: paksungwoo@naver.com; 2 Department of Architectural Engineering, Yonsei University, Seoul 120-749, Korea; E-Mail: aeioobk@yonsei.ac.kr

**Keywords:** average strain, vibrating wire strain gauge, maximum stress, structural health monitoring

## Abstract

The safety of a multi-span waler beam subjected simultaneously to a distributed load and deflections at its supports can be secured by limiting the maximum stress of the beam to a specific value to prevent the beam from reaching a limit state for failure or collapse. Despite the fact that the vast majority of accidents on construction sites occur at waler beams in retaining wall systems, no safety monitoring model that can consider deflections at the supports of the beam is available. In this paper, a maximum stress estimation model for a waler beam based on average strains measured from vibrating wire strain gauges (VWSGs), the most frequently used sensors in construction field, is presented. The model is derived by defining the relationship between the maximum stress and the average strains measured from VWSGs. In addition to the maximum stress, support reactions, deflections at supports, and the magnitudes of distributed loads for the beam structure can be identified by the estimation model using the average strains. Using simulation tests on two multi-span beams, the performance of the model is evaluated by estimating maximum stress, deflections at supports, support reactions, and the magnitudes of distributed loads.

## 1. Introduction

Safety monitoring of multi-span beam structures under distributed loads has been an issue of interest since structural health monitoring (SHM) has been introduced [1,2,3,4,5,6,7,8]. If the maximum stress in a beam structure exceeds the specified strength or allowable stress due to unexpected loads, then it can be considered that the safety of the beam structure has reached a limit state. Therefore, structural responses, including the maximum stress in beam structures must be monitored in the long term to assess the safety of a structure [9,10,11,12,13].

To estimate the maximum strain or stress of multi-span beams, mathematical models based on measured average strains have been developed and applied to monitoring the safety of beam structures [14]. In the mathematical models, the maximum strain of a multi-span beam is obtained by defining the relationship between the average strain measured from sensors and the estimated maximum strain. It is shown that the maximum strain of a multi-span beam can be accurately estimated by using average strains obtained from long gauge fiber optic sensors (LGFOS) or vibrating wire strain gauges (VWSG) [15,16,17,18].

**Figure 1:**
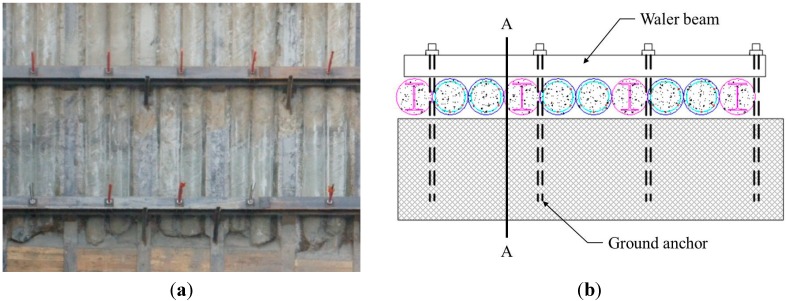
A multi-span waler beam supported by ground anchors in a retaining wall system: (**a**) Front view; (**b**) Side view.

However, for the case of beam structures subjected to deflections at the supports, the mathematical models of multi-span beam without consideration of the effect of deflections at supports on the magnitude of the maximum stress cannot be used in the assessment of the safety of beam structures. A typical example of a beam structure subjected to deflections at its supports is the waler beam in an anchored retaining wall system shown in Figure 1. The waler beam is used to transfer retained loads due to lateral pressures from the retained soil evenly between the ground anchors [19]. 

**Figure 2:**
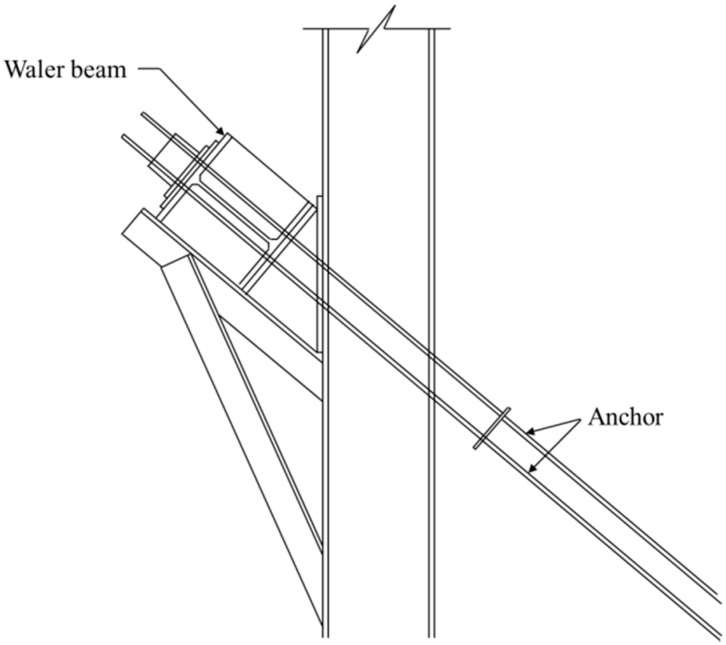
Section A-A of a waler beam supported by ground anchors in a retaining wall system.

As shown in Figure 2, the ground anchors in a retaining wall can be modelled as supports for the waler beam. Since the ground anchors are subjected to the axial tension due to lateral soil pressure, axial displacement along the axis of the ground anchor occurs and then the waler beam supported by the ground anchors is subjected to differential support deflections. Excessive structural deformation in waler beams due to lateral pressure and differential support deflections is a main cause of damage or collapse of retaining wall systems [20]. The safety of the retaining wall system can be assessed by monitoring the safety of the waler beam subjected to deflections at the supports. Thus, it is necessary to develop a generalized mathematical model for estimation of the maximum stress in a multi-span beam subjected to support deflections.

Therefore, in this paper, an analytical model to estimate the maximum stress for a multi-span beam with deflections of supports under distributed loads is presented. For the estimation, the relationship between the maximum stress and the average strain of each span is derived and the average strain is measured at the middle of each span. In addition to estimation of the maximum stress, using average strains from VWSGs, estimation models for deflections at supports, support reactions, and the magnitudes of distributed loads are presented. For the safety monitoring of multi-span beam structures in retaining wall systems, the model is applied to estimation of the maximum stress and distribution of moments along the length of a beam.

## 2. Average Strain of Waler Beams Measured by VWSGs

**Figure 3:**
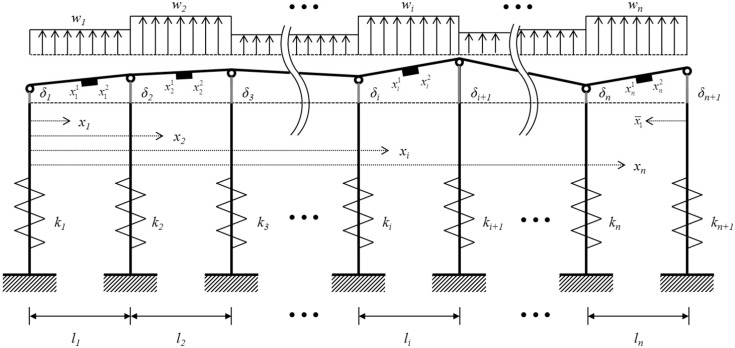
*n*-Span continuous beam supported by *n +* 1 ground anchors.

In Figure 3, a typical waler beam supported by *n +* 1 ground anchors in a retaining wall system is modeled a linear elastic *n*-span continuous beam with *n +* 1 supports. $l_{i}$ and $w_{i}$ are the length and the magnitude of distributed lateral load of the *i*th span, respectively. $k_{i}$ and $\delta_{i}$ are the stiffness of the anchor and the deflection at the *i*th supports, respectively. It is assumed that the deflection at each support has a different value.

For a linear elastic multi-span beam as shown in Figure 3, the relationship between the bending stress, $\sigma (x_{i})$, and strain, $\varepsilon (x_{i})$, as a function of the position of $x_{i}$ in the *i*th span along the axis of the beam can be written as:

(1) $$\sigma (x_{i})=\frac{M(x_{i})}{Z}=\varepsilon (x_{i})E$$

where $M(x_{i})$ is the bending moment, Z is the elastic section modulus and E is the modulus of elasticity of the beam [21]. Using the predetermined value for E, the stress in Equation (1) can be estimated by measuring the strain $\varepsilon (x_{i})$. For the measurement of strains, VWSGs are the most frequently used sensors in the construction field of building structures since operating principle is simple and installation cost is relatively low compared to other sensors [16,17]. In addition, VWSGs have excellent endurance and are free from electromagnetic interference (EMI) so that VWSGs suitable for long-term monitoring in the field [22,23,24].

**Figure 4:**
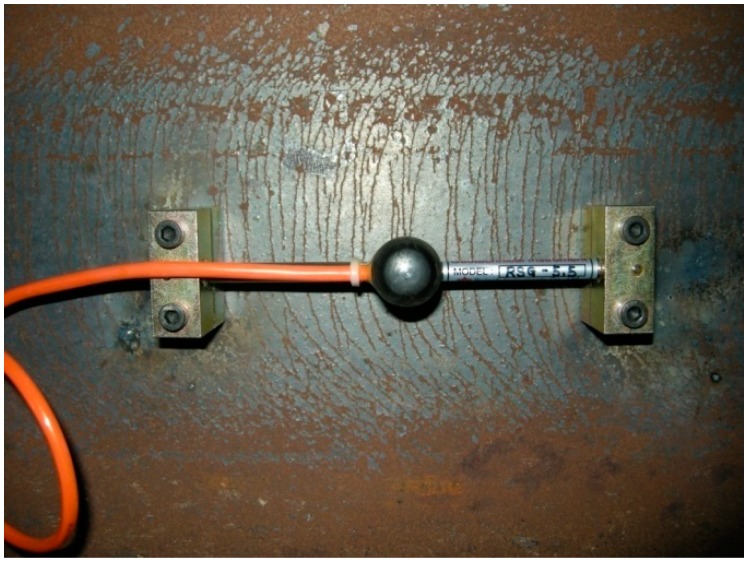
Typical vibrating wire strain gauge attached to the surface of a steel section for measurement of average strains.

As shown in Figure 4, a VWSG consists of three components of two mounting blocks which are attached to the surface of a structural member to be measured, a vibrating wire whose frequency changes due to tension or compression between the two mounting blocks, and plucking and pickup coil, which excited the vibrating wire and measure its resonance frequencies. The change of length of a vibrating wire which is fixed at the two mounting blocks causes the variation of the natural frequency of the wire. For this reason, a VWSG measures the average strain over the gauge length between the two mounting blocks. As shown in Figure 3, if the gauge length is the distance between the mounting blocks and the locations of the mounting blocks for the VWSG in the *i*th span of the waler beam are set to $x_{i}^{1}$ and $x_{i}^{2}$, then the average strain for the *i*th span of the waler beam, $\varepsilon_{avg,i}$, can be given as:

(2) $$\varepsilon_{avg,i}=\frac{\int_{x_{i}^{1}}^{x_{i}^{2}}\varepsilon (x_{i})dx}{x_{i}^{2}-x_{i}^{1}}$$

Then, the relationship between the bending moment at the *i*th span, $M(x_{i})$ in Equation (1) and the measured average strain $\varepsilon_{avg,i}$ in Equation (2) can be expressed by:

(3) $$\varepsilon_{avg,i}=\frac{\int_{x_{i}^{1}}^{x_{i}^{2}}M(x_{i})dx}{EZ(x_{i}^{2}-x_{i}^{1})}$$

## 3. Estimation Model for Maximum Stress

Based on the three moment theorem, the relationship among the bending moments, $M_{i-1}$, $M_{i}$ and $M_{i+1}$, at three consecutive supports of the multi-span beam in Figure 3 is given by Equation (A1) in Appendix A. The beam is assumed to have constant moment of inertia and elastic modulus. For the continuous beam with *n +* 1 supports, *n −* 1 three moment equations can be formulated using Equation (A1) in Appendix A and expressed in matrix form as:

(4) [A]{M}=[B]{w}+[C]{δ}

where [A] is a square matrix of dimensions (n−1)×(n−1), [B] is a matrix of dimensions (n−1)×n, and [C] is a matrix of dimensions (n−1)×(n+1), given by Equations (B1)–(B3) in Appendix B. Also, {*M*}, {*w*} and {δ} are the bending moment vector of dimension (n−1) ×1, the magnitude of distributed lateral load vector of dimension n×1, and the deflection at support vector of dimension (n+1)×1, respectively. Then, the equations in Equation (4) can be solved simultaneously for the moments at supports. Having found the moments at *n +* 1 supports, the vector of dimension (n+1)×1 for the reactions at the supports of the continuous beam, {R}, can be found by:

(5) $$\left\{R\}=[G]\{w\}+[C^{\prime }]\{M\right\}$$

where [G] is a matrix of dimensions (n−1)×n and [*C′*] is a matrix of dimensions (n+1)×(n−1), given by Equations (B4) and (B5) in Appendix B. As shown in Figure 3, the reaction at the *ith* support can also be found by multiplying the stiffness *k_i_* by the deflection δ*_i_* of the support. Then, the reaction vector in Equation (5) can be expressed by substituting the moment vector {*M*} in Equation (4) into Equation (5).

(6) $$\{R\}=[K]\{\delta \}=[G]\{w\}+[C^{\prime }]\left[\left[A^{-1}B]\{w\}+[A^{-1}C]\{\delta \right\}\right]$$

Where [*K*] is the stiffness matrix for the *n +* 1 supports in Figure 3. From Equation (6), the relationship between the vectors {*w*} and {δ} is found by:

(7) $$\left[G+C^{\prime }A^{-1}B]\{w\}=[K-C^{\prime }A^{-1}C]\{\delta \right\}$$

Using the support reactions {*R*} and distributed lateral loads {*w*}, the bending moment of each span of the beam $\left\{\bar{M}\left(x\right)\right\}$ can be given by:

(8) $$\left\{\bar{M}\left(x\right)\right\}=[P]\{R\}-[Q]\{w\}$$

where [P] is a matrix of dimensions (n+1)×(n+1) and [Q] is a matrix of dimensions (n+1)×1, given by Equations (B6) and (B7) in Appendix B.

By considering the gauge length of a VWSG in Equation (3), the relationship in Equation (8) for the *i*th span can be evaluated by numerical integration over the gauge length of $x_{i}^{2}-x_{i}^{1}$ as below:

(9) $$\left\{\int_{x_{i}^{1}}^{x_{i}^{2}}\bar{M}\left(x\right)\;dx\right\}=[\bar{P}]\{R\}-[\bar{Q}]\{w\}$$

where $\left[\bar{P}\right]$ and $\left[\bar{Q}\right]$ are integrals of [P] and [Q] in Equations (B6) and (B7) in Appendix B over the gauge length, respectively. The integral of the moment over the interval of the gauge length in Equation (9) can be expressed in term of the average strain in Equation (3):

(10) $$\left\{\int_{x_{i}^{1}}^{x_{i}^{2}}\bar{M}\left(x\right)\;dx\right\}=\left\{\varepsilon_{avg,i}\right\}EZ(x_{i}^{2}-x_{i}^{1})=[\bar{P}]\{R\}-[\bar{Q}]\{w\}$$

Then, by substituting {R} in Equation (6) and {w} in Equation (7) into Equation (10), Equation (10) can be given by:

(11) $$EZ(x_{i}^{2}-x_{i}^{1})\left\{\varepsilon_{avg,i}\right\}=[\bar{P}][K]\left\{\delta \right\}-[\bar{Q}]{\left[{\left[G+C^{\prime }A^{-1}B\right]}^{T}[G+C^{\prime }A^{-1}B]\right]}^{-1}{\left[G+C^{\prime }A^{-1}B\right]}^{T}[K-C^{\prime }A^{-1}C]\{\delta \}$$

From Equation (11), deflections at supports of the waler beam {δ} can be found from the measurement of average strains from VWSGs $\left\{\varepsilon_{avg,i}\right\}$ as below:

(12) $$\left\{\delta \}=EZ(x_{i}^{2}-x_{i}^{1}){\left[\left[\bar{P}][K]+[\bar{Q}]{\left[{\left[\bar{Y}\right]}^{T}[\bar{Y}]\right]}^{-1}{\left[\bar{Y}\right]}^{T}[\bar{X}\right]\right]}^{-1}\{\varepsilon_{avg,i}\right\}$$

where $\left[\bar{X}]=[K-C^{\prime }A^{-1}C\right]$ and $\left[\bar{Y}]=[G+C^{\prime }A^{-1}B\right]$. Thus, by substituting the deflections determined from the average strains in Equation (12) into Equations (6) and (7), reaction at each support {R} and the magnitude of the distributed lateral load at each span {w} can be identified. Consequently, the bending moment of each span can be determined by substituting {R} and {δ} into Equation (9). Finally, based on the relationship in Equation (1), the maximum stress of an *n*-span waler beam can be determined from the average strains measured by VWSGs.

## 4. Test of the Model on Multi-Span Beams

### 4.1. Four-Span Beam

**Figure 5:**
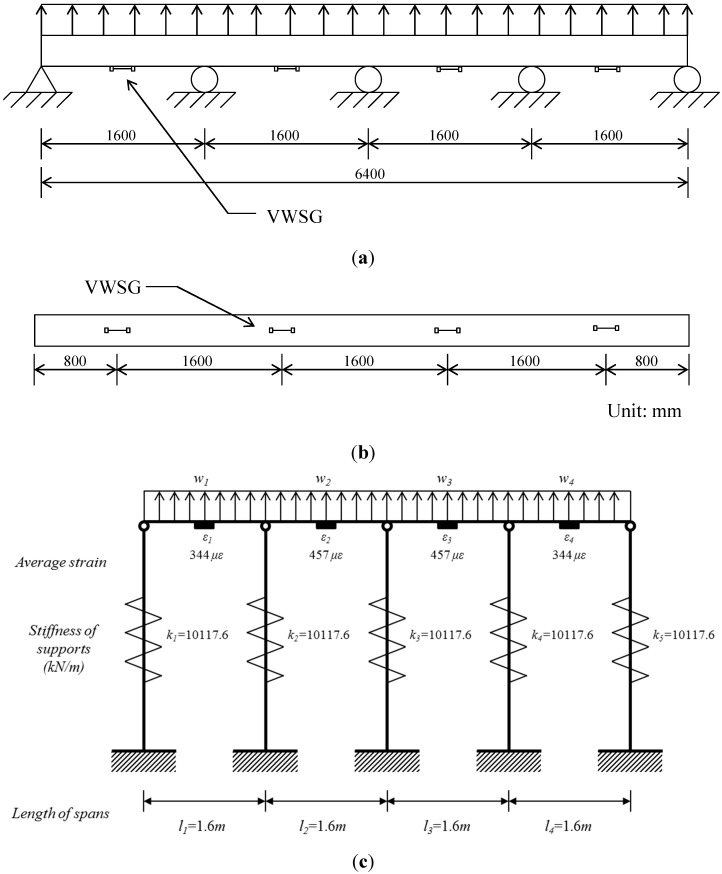
Experimental setup for the test of 4-span beam: (**a**) Side view; (**b**) Bottom view; (**c**) Conceptual model.

The setup for simulation of the four-span test beam is shown in Figure 5. The section of beam is H-300 × 300 × 10 × 15, with a depth of 300 mm, a flange width of 300 mm, a web thickness of 10 mm, and a flange thickness of 15 mm. The material for the beam section is SS400 grade steel with a modulus of elasticity of 205 GPa and yield strength of 235.3 MPa. The test model is designed to simulate the collapse of the waler beam in a retaining wall at a construction site in 2009. The waler beam in the retaining wall was designed to carry a uniformly distributed load of 206.5 kN/m. The waler beam was supported by ground anchors of prestressed concrete (PC) strands of Φ12.7 mm × 4 at a horizontal spacing of 1.6 m. The axial stiffness of the anchor was given by 10,117.6 kN/m. With these properties, the four-span beam in the construction site can be modeled as a conceptual model in Figure 5c.

To test the conceptual model, it is assumed that the vibrating wire strain gauges with the gauge length of 15 cm are installed to measure the average strains in Figure 5b. As an input, the measured average strains are assumed to be ε_1_ = ε_4_ = 344 με and ε_2_ = ε_3_ = 457 με. For the given values for average strains obtained from the numerical analysis with the properties of the model in Figure 5c, the magnitudes of deflections of supports, reactions, and distributed loads in Table 1 are estimated from the analytical model in Equations (6), (7) and (12), respectively.

**Table 1:** Estimated values for deflections, reactions, and distributed loads for the 4-span beam.

Deflection (m)	Reaction (kN)	Distributed load (kN/m)
$\delta_{1}$ = 0.0201	R_1_ = 202.9	*w*_1_ = 206.5
$\delta_{2}$ = 0.0291	R_2_ = 294.3	*w*_2_ = 206.5
$\delta_{3}$ = 0.0323	R_3_ = 327.2	*w*_3_ = 206.5
$\delta_{4}$ = 0.0291	R_4_ = 294.3	*w*_4_ = 206.5
$\delta_{5}$ = 0.0201	R_5_ = 202.9	

**Figure 6:**
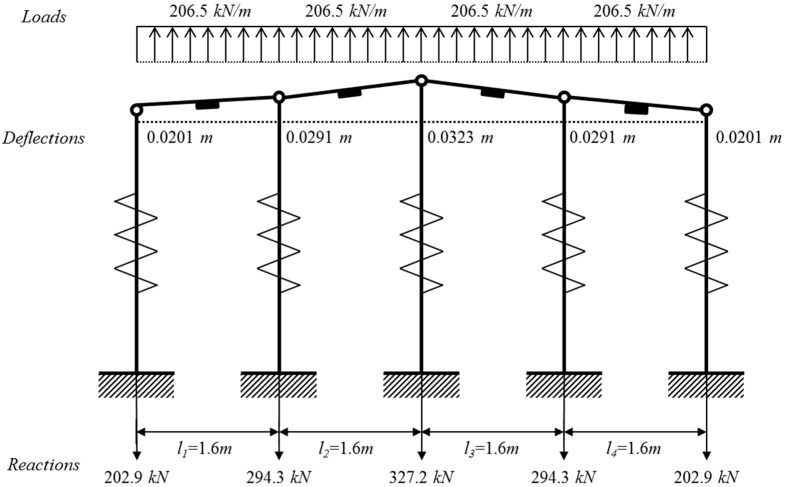
Deformed shape of 4-span beam subjected to the distributed lateral loads.

The deformed shape based on estimated support deflections, support reactions, and applied loads of the test beam is shown in Figure 6. As can be seen in Figure 6, a maximum deflection of 3.23 cm and a maximum reaction of 327.2 kN were calculated at the third support. 

**Figure 7:**
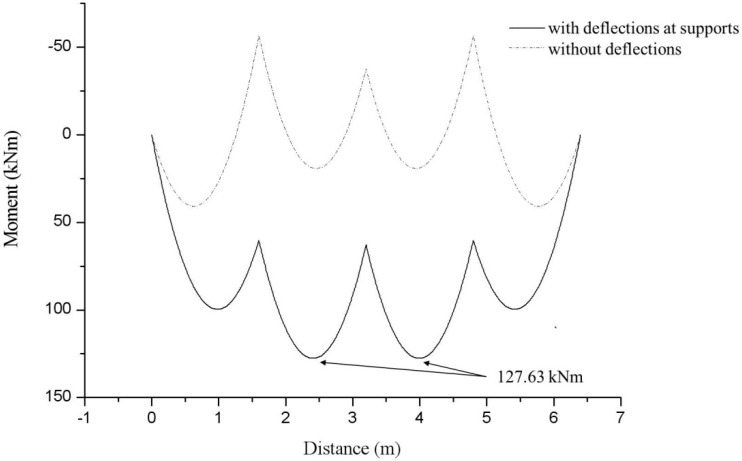
Bending moment diagram for a 4-span beam for estimation of maximum stress.

In Figure 7, the bending moment diagram with the consideration of support deflections are compared with the bending moment distribution without the consideration of deflections at the supports. As can be seen in Figure 7, maximum bending moments of 127.63 kNm were estimated at the points of 2.4 m and 3.93 m measured from the left end of the beam. Using the modulus of elasticity of 205 GPa in Equation (1), maximum stresses of 93.85 MPa were estimated at the same points of the four-span beam where the maximum moment occurs. Thus, for a given value of the average strains measured by VWSGs, it is confirmed that the reactions, distributed loads, bending moments, and maximum stress of the 4-span test beam can be estimated by the proposed analytical model.

As a practical application of the analytical model presented in the paper, the measured values of the average strain can be limited to a specific value to prevent the waler beam from reaching one of limit states for failure or collapse of waler beams. For the safety monitoring of the four-span waler beam, the average strains at the second and third spans, ε_2_ and ε_3_, may be limited to the value of 780 με to prevent the waler beam from reaching the allowable stress of 160 MPa for SS400 grade steel beam used in the construction site.

### 4.2. Eight-Span Beam

**Figure 8:**
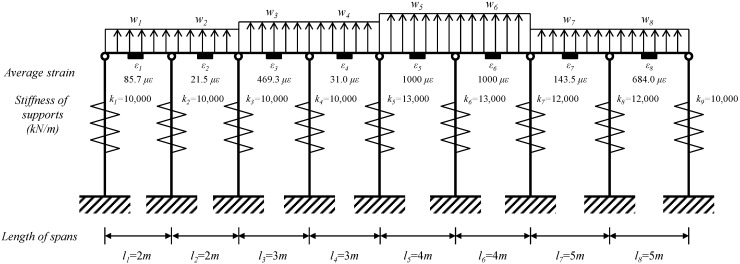
Schematic diagram for the 8-span beam with irregularities in geometry, mechanical properties, and magnitudes of lateral loads.

A schematic diagram for the eight-span test beam is shown in Figure 8. The material for the beam section of H-300 × 300 × 10 × 15 is SS400 grade steel with a modulus of elasticity of 205 GPa and yield strength of 235.3 MPa. To test the applicability of the estimation method, the test beam in Figure 8 is designed to have irregularities in geometry, mechanical properties of anchors, and the magnitudes of distributed lateral loads.

The waler beam was designed to carry three different magnitudes of distributed design loads of *w*_1_ = *w*_2_ = *w*_7_ = *w*_8_ = 100 kN/m, *w*_3_ = *w*_4_ = 200 kN/m, and *w*_5_ = *w*_6_ = 300 kN/m. As input for the estimation model in Equation (12), the average strains from VWSGs are listed in Table 2. For the given values for average strains obtained from the numerical analysis with the properties of the model in Figure 8, the magnitudes of deflections of supports, reactions, and distributed loads in Table 3 are estimated from the analytical model in Equations (6), (7) and (12), respectively.

**Table 2:** Span length, stiffness of anchor, and average strain for an 8-span beam.

Span Length (m)	Stiffness of Anchor (kN/m)	Average Strain (με)
$l_{1}$ = 2.0	$k_{1}$ = 10,000.0	$\varepsilon_{1}$ = 85.7
$l_{2}$ =2.0	$k_{2}$ = 10,000.0	$\varepsilon_{2}$ = 21.5
$l_{3}$ = 3.0	$k_{3}$ = 10,000.0	$\varepsilon_{3}$ = 469.3
$l_{4}$ = 3.0	$k_{4}$ = 10,000.0	$\varepsilon_{4}$ = 31.0
$l_{5}$ = 4.0	$k_{5}$ = 13,000.0	$\varepsilon_{5}$ = 1,000.0
$l_{6}$ = 4.0	$k_{6}$ = 13,000.0	$\varepsilon_{6}$ = 1,000.0
$l_{7}$ = 5.0	$k_{7}$ = 12,000.0	$\varepsilon_{7}$ = 143.5
$l_{8}$ = 5.0	$k_{8}$ = 12,000.0	$\varepsilon_{8}$ = 684.0
	$k_{9}$ = 10,000.0	

**Table 3:** Estimated deflections, reactions, and magnitudes of distributed loads for an eight-span beam.

Deflection (cm)	Reaction (kN)	Distributed Load (kN/m)
$\delta_{1}$ = 3.01	R_1_ = 75.3	*w*_1_ = 100
$\delta_{2}$ = 8.88	R_2_ = 222.0	*w*_2_ = 100
$\delta_{3}$ = 14.83	R_3_ = 378.8	*w*_3_ = 200
$\delta_{4}$ = 23.23	R_4_ = 586.6	*w*_4_ = 200
$\delta_{5}$ = 31.85	R_5_ = 995.5	*w*_5_ = 300
$\delta_{6}$ = 42.92	R_6_ =1287.7	*w*_6_ = 300
$\delta_{7}$ = 47.49	R_7_ = 712.4	*w*_7_ = 100
$\delta_{8}$ = 38.38	R_8_ = 575.7	*w*_8_ = 100
$\delta_{9}$ = 22.00	R_9_ = 220.0	

**Figure 9:**
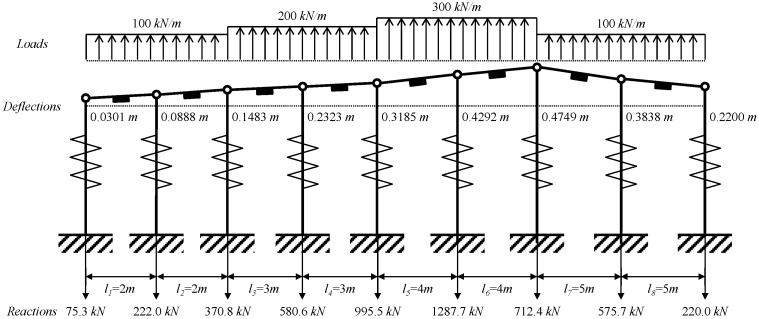
Deformed shape of an 8-span beam subjected to distributed lateral loads.

**Figure 10:**
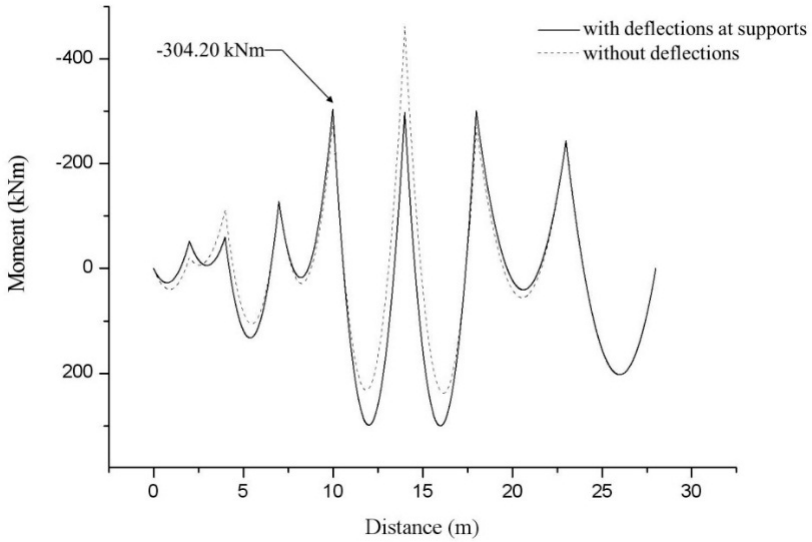
Bending moment diagram for an 8-span beam for estimation of maximum stress.

The deformed shape based on estimated support deflections, support reactions, and applied loads of the test beam are shown in Figure 9. As can be seen in Figure 9, maximum deflection of 47.5 cm and maximum reaction of 1287.7 kN were calculated at the 7th and 6th supports, respectively. In Figure 10, the bending moment diagram with the consideration of support deflections are compared with the bending moment distribution without the consideration of deflections at the supports. As can be seen in Figure 10, a maximum negative bending moment of −304.20 kNm was estimated at the 5th support. Using the modulus of elasticity of 205 GPa, a maximum stress of −223.67 MPa was estimated at the same points where the maximum moment occurs, therefore, it is found that the magnitude of the estimated maximum stress exceeded the allowable stress of 160 MPa for a SS400 grade steel beam.

## 5. Conclusions

In this paper, an analytical model for estimation of the maximum stress of a multi-span beam with deflections of its supports is presented. The analytical model is derived by defining the relationship between the maximum stress and the average strain of each span of the multi-span beam. Based on the use of the average strains obtained from VWSGs as the input to the analytical model, the analytical model allows estimation of deflections at supports, support reactions, and the magnitudes of distributed loads. Using tests on two multi-span beams, the performance of the model is evaluated by estimating maximum stresses, support deflections, support reactions, and the magnitudes of distributed loads. From the simulation test, it can be concluded that safety monitoring of multi-span waler beams is made possible by comparing the given allowable stress of the beam with the estimated maximum stress with consideration of any deflections at the supports. Through periodic estimation of the maximum stress by the proposed method, the safety of retaining walls at construction sites will be improved.

## Appendix

### A. Three Moment Equation

(A1) $$M_{i}l_{i}+2M_{i+1}(l_{i}+l_{i+1})+M_{i+2}l_{i+1}=-\frac{1}{4}\left(w_{i}l_{i}^{3}+w_{i+1}l_{i+1}^{3}\right)+6EI\left(-\frac{1}{l_{i}}\left(\delta_{i}\right)+\left(\frac{1}{l_{i}}+\frac{1}{l_{i+1}}\right)\left(\delta_{i+1}\right)-\frac{1}{l_{i+1}}\left(\delta_{i+2}\right)\right)$$

where $M_{i}$, $l_{i}$, $w_{i}$ and $\delta_{i}$ are the bending moment, length, distributed lateral load and deflection at the *i*th support of the beam, respectively. E and I are elastic modulus and moment of inertia, respectively.

### B. Matrices in Section 3

(B1) $$[A]=\left(\begin{array}{cccccc} 2(l_{1}+l_{2}) & l_{2} & 0 & \cdots & \cdots & 0\\ l_{2} & 2(l_{2}+l_{3}) & l_{3} & 0 & \cdots & \vdots \\ 0 & l_{3} & 2(l_{3}+l_{4}) & l_{4} & 0 & \vdots \\ \vdots & 0 & \ddots & \ddots & \ddots & 0\\ \vdots & \vdots & 0 & l_{n-2} & 2(l_{n-2}+l_{n-1}) & l_{n-1}\\ 0 & \cdots & \cdots & 0 & l_{n-1} & 2(l_{n-1}+l_{n}) \end{array}\right)$$

(B2) $$[B]=-\frac{1}{4}\left(\begin{array}{ccccc} l_{1}^{3} & l_{2}^{3} & 0 & \cdots & 0\\ 0 & l_{2}^{3} & l_{3}^{3} & \ddots & \vdots \\ \vdots & \ddots & \ddots & \ddots & 0\\ 0 & \cdots & 0 & l_{n-1}^{3} & l_{n}^{3} \end{array}\right)$$

(B3) $$[C]=6EI\left(\begin{array}{cccccc} -\frac{1}{l_{1}} & \frac{1}{l_{1}}+\frac{1}{l_{2}} & -\frac{1}{l_{2}} & 0 & \cdots & 0\\ 0 & -\frac{1}{l_{2}} & \frac{1}{l_{2}}+\frac{1}{l_{3}} & -\frac{1}{l_{3}} & \ddots & \vdots \\ \vdots & \ddots & \ddots & \ddots & \ddots & 0\\ 0 & \cdots & 0 & -\frac{1}{l_{n-1}} & \frac{1}{l_{n-1}}+\frac{1}{l_{n}} & -\frac{1}{l_{n}} \end{array}\right)$$

(B4) $$[G]=\frac{1}{2}\left(\begin{array}{ccccc} l_{1} & 0 & \cdots & \cdots & 0\\ l_{1} & l_{2} & 0 & \cdots & \vdots \\ 0 & l_{2} & l_{3} & 0\cdots & \vdots \\ \vdots & 0 & \ddots & \ddots & 0\\ \vdots & \vdots & 0 & l_{n-1} & l_{n}\\ 0 & \cdots & \cdots & 0 & l_{n} \end{array}\right)$$

(B5) $$[C^{\prime }]=-\frac{1}{6EI}{\left[C\right]}^{T}$$

(B6) $$[P]=\left(\begin{array}{cccccc} x_{1} & 0 & \cdots & \cdots & 0 & 0\\ x_{2} & x_{2}-l_{1} & 0 & \cdots & \ddots & \vdots \\ \vdots & \vdots & x_{3}-l_{1}-l_{2} & 0 & \vdots & \vdots \\ \vdots & \vdots & \vdots & \ddots & 0 & \vdots \\ x_{n} & x_{n}-l_{1} & x_{n}-l_{1}-l_{2} & \cdots & x_{n}-l_{1}-\cdots -l_{n-1} & 0\\ 0 & \cdots & \cdots & 0 & 0 & {\bar{x}}_{0} \end{array}\right)$$

(B7) $$[Q]=\left(\begin{array}{cccccc} \frac{1}{2}x_{1}^{2} & 0 & & & & \\ l_{1}(x_{2}-\frac{l_{1}}{2}) & \frac{1}{2}x_{2}^{2} & 0 & \cdots & \cdots & 0\\ l_{1}(x_{3}-\frac{l_{1}}{2}) & l_{2}(x_{3}-l_{1}-\frac{l_{2}}{2}) & \frac{1}{2}x_{3}^{2} & 0 & \ddots & \vdots \\ \vdots & \vdots & \vdots & \ddots & 0 & \vdots \\ l_{1}(x_{n}-\frac{l_{1}}{2}) & l_{2}(x_{n}-l_{1}-\frac{l_{2}}{2}) & \cdots & & l_{n-1}(x_{n}-l_{1}-\cdots l_{n-2}-\frac{l_{n-1}}{2}) & 0\\ 0 & \cdots & \cdots & \cdots & 0 & \frac{1}{2}{\bar{x}}_{0}^{2} \end{array}\right)$$

In Equations (B6) and (B7), $x_{i}$ ($l_{1}+\cdots +l_{n-1}\leq x_{i}<l_{1}+\cdots +l_{n}$) is the distance from the left end of the beam, as shown in Figure 3, and ${\bar{x}}_{0}$ ($0\leq {\bar{x}}_{0}<l_{n}$) is the distance from the right side of the beam to the position in the last span.
